# Bayesian Networks in the Management of Hospital Admissions: A Comparison between Explainable AI and Black Box AI during the Pandemic †

**DOI:** 10.3390/jimaging10050117

**Published:** 2024-05-10

**Authors:** Giovanna Nicora, Michele Catalano, Chandra Bortolotto, Marina Francesca Achilli, Gaia Messana, Antonio Lo Tito, Alessio Consonni, Sara Cutti, Federico Comotto, Giulia Maria Stella, Angelo Corsico, Stefano Perlini, Riccardo Bellazzi, Raffaele Bruno, Lorenzo Preda

**Affiliations:** 1 Department of Electrical, Computer and Biomedical Engineering, University of Pavia, 27100 Pavia, Italy; giovanna.nicora@unipv.it (G.N.); riccardo.bellazzi@unipv.it (R.B.); 2 Diagnostic Imaging and Radiotherapy Unit, Department of Clinical, Surgical, Diagnostic and Pediatric Sciences, University of Pavia, 27100 Pavia, Italy; michele.catalano01@universitadipavia.it (M.C.); marinafrancesc.achilli01@universitadipavia.it (M.F.A.); gaia.messana01@universitadipavia.it (G.M.); antonio.lotito01@universitadipavia.it (A.L.T.); alessio.consonni01@universitadipavia.it (A.C.); lorenzo.preda@unipv.it (L.P.); 3 Radiology Institute, Fondazione IRCCS Policlinico San Matteo, 27100 Pavia, Italy; 4 Medical Direction, Fondazione IRCCS Policlinico San Matteo, 27100 Pavia, Italy; s.cutti@smatteo.pv.it; 5 Reply S.p.A. Corso Francia, 110, 10143 Turin, Italy; f.comotto@reply.it; 6 Department of Internal Medicine and Therapeutics, University of Pavia, 27100 Pavia, Italy; giuliamaria.stella@unipv.it (G.M.S.); angelo.corsico@unipv.it (A.C.); stefano.perlini@unipv.it (S.P.); 7 Unit of Respiratory Diseases, Fondazione IRCCS Policlinico San Matteo, 27100 Pavia, Italy; 8 Department of Emergency, Fondazione IRCCS Policlinico San Matteo, 27100 Pavia, Italy; 9 Department of Clinical, Surgical, Diagnostic and Pediatric Sciences, University of Pavia, 27100 Pavia, Italy; raffaele.bruno@unipv.it; 10 Unit of Infectious Diseases, Fondazione IRCCS Policlinico San Matteo, 27100 Pavia, Italy

**Keywords:** artificial intelligence, explainability, machine learning, Random Forest, Bayesian Networks, COVID-19

## Abstract

Artificial Intelligence (AI) and Machine Learning (ML) approaches that could learn from large data sources have been identified as useful tools to support clinicians in their decisional process; AI and ML implementations have had a rapid acceleration during the recent COVID-19 pandemic. However, many ML classifiers are “black box” to the final user, since their underlying reasoning process is often obscure. Additionally, the performance of such models suffers from poor generalization ability in the presence of dataset shifts. Here, we present a comparison between an explainable-by-design (“white box”) model (Bayesian Network (BN)) versus a black box model (Random Forest), both studied with the aim of supporting clinicians of Policlinico San Matteo University Hospital in Pavia (Italy) during the triage of COVID-19 patients. Our aim is to evaluate whether the BN predictive performances are comparable with those of a widely used but less explainable ML model such as Random Forest and to test the generalization ability of the ML models across different waves of the pandemic.

## 1. Introduction

Artificial Intelligence (AI) and Machine Learning (ML) approaches are generally recognized as useful tools to support clinicians in their decision process. AI/ML tools have been developed to solve various medical problems, for instance, to predict disease diagnosis [1], to support cancer detection in medical images [2], to detect Alzheimer’s from webcam gaze trackers [3], and even to determine the best treatment based on patient’s clinical and genomic characteristics [4,5]. As research studies examining AI/ML applications in medicine have been growing over the years, interest has raised in the actual implementation of these systems into clinical practice [6]. The recent SARS-CoV-2 pandemic has spurred an acceleration of digital health technologies and AI/ML implementation to combat the pandemic, ranging from supporting hospital admissions to defining new therapeutic strategies [7,8]. Nevertheless, building Machine Learning (ML) approaches that can be generalized over time and/or on patients coming from different hospitals is challenging. Since ML relies on data for training, the less the training data are representative of the true underlying population, the less reliable the ML model will be when applied to new data that may deviate from the training population. As a matter of fact, ML inherently suffers from dataset shifts and poor generalization ability across different populations [9,10,11], which leads to a decrease in trust in AI/ML predictions. For instance, a recent paper showed the impact of data drift on the performance of AI models designed to predict sepsis, advocating for frequent re-training of such models [12].

The pandemic has been a perfect testing ground since several sources of data shifts have occurred. Several SARS-CoV-2 variants have arisen, from the Alpha variant recognized as a Variant of Concern (VOC) in late December 2020 to the Omicron variant dominating the virus landscape from autumn 2021. Each VOC exhibits different characteristics, such as increased transmissibility, reduction of treatment and vaccine efficacy, and severity of symptoms. As our clinical and biological knowledge increased, new treatment protocols were defined over the pandemic. Consequently, AI/ML models trained on data collected at particular time intervals may not be calibrated to be used on data collected subsequently, and a re-training strategy should be defined. For instance, in [13], we developed an ML model to predict new SARS-CoV-2 variants of concern throughout the pandemic based on the Spike protein sequence. We simulated the implementation of this algorithm from the beginning of the pandemic to March 2023 and we periodically re-trained the model whenever the World Health Organization (WHO) recognized a new variant as a Variant of Concern.

Another aspect that undermines trust in AI/ML is the perception that the AI/ML classification process is obscure. Widely used machine learning algorithms from Deep Learning to Tree Ensembles enable rapid learning with high performance from extensive data sources, but the reasoning process between input and output variables is usually a black box to the user. Guidotti et al. defined a black box as “any data mining or machine learning obscure model, whose internals are either unknown to the observer or they are known but uninterpretable by human” [14]. Following this definition, deep learning algorithms, as well as other machine learning tools, such as Random Forests and other ensembles, are considered as black boxes. Interestingly, the degree of interpretability is not only related to the intrinsic characteristics and structure of a machine learning model, but also to its complexity, as even some inherently interpretable models, such as decision trees, quickly become less interpretable as their complexity increases [14,15].

However, intelligent systems should be able to explain their reasoning to human users, thus improving human–AI interaction [16] and promoting human-in-the-loop (HITL), human-on-the-loop (HOTL), and human-in-command (HIC). These three approaches delineate the capability of human intervention during the ML design cycle and monitoring of the activity, and they are also underlined in the Ethics Guidelines for Trustworthy AI [17], which laid the foundation of the recently proposed AI act, the first comprehensive regulation of AI promoted by the European Union. As AI medical applications are identified as “high risk” according to this regulation, AI tools to be applied in healthcare will be required to fulfill transparency and interpretability requirements, allowing users, such as clinicians, to follow the AI reasoning process for every single prediction.

Yet, black box models are widely applied as they are often perceived as better performing in comparison with “white box”, or “explainable-by-design” Machine Learning approaches, such as Logistic Regression or Bayesian Networks. Such models do not need any additional layers to be interpreted by humans, but their predictions can be easily understood by humans. Current research on AI Explainability (XAI) aims to make black box ML predictions more transparent and explainable. In this direction, different XAI approaches have been developed. Many of these tools provide explanations of single ML predictions by highlighting the important features, i.e., characteristics of the output, that lead the classifier to its final decision [18]. Other XAI approaches approximate the complex model with a “white box” model on a local neighborhood of the prediction that needs to be explained [19]. However, as recently stated, in order to reach explainability in medicine we need also to promote causability. Causability refers to the extent to which an explanation achieves a specified level of causal understanding in a specific context of use [20]. Additionally, popular XAI methods, such as LIME and SHAP, have been shown to be unreliable in the case of adversarial attacks, where the goal is to cause an AI to make a misclassification by manipulating the input data [21]. Explanations derived from current XAI methods provide spurious correlations rather than cause/effect relationships, leading to erroneous or even biased explanations [22]. A recent study compared an explainable-by-design model (or “white box”), namely, a Bayesian Network (BN), with the explanations derived from XAI methods, by performing a survey with human participants. They found that BNs are easier to interpret compared to XAI methods [23]. Researchers have also emphasized that the belief in black box models surpassing explainable-by-design models in performance is unfounded and that XAI explanations may not consistently reflect the original model’s computations. Therefore, they propose that, in high-stakes applications such as healthcare, AI developers should refrain from employing black box models and their corresponding explainers. Instead, authors advocate for the development of models inherently interpretable from their inception [24]. 

In the context of predicting hospital admission for COVID-19 through artificial intelligence and machine learning (AI/ML), we have a dual objective. The first objective is to explore the impact of data drift on the performance of a model developed during the first wave of the COVID-19 pandemic. Data drift refers to any change in data over time that could affect the performance of the model. This is particularly relevant in the context of COVID-19, where the characteristics of the disease, treatment strategies, and health resources have changed over time. The second objective is to quantify whether an explainable-by-design model, such as a Bayesian Network (BN), has similar performance compared to a more complex, black box model, as defined above.

In particular, we developed AI/ML models that suggest to doctors whether patients with COVID-19 in triage could be treated at home or need to be hospitalized. This is a critical task, as effective management of patients with COVID-19 can have a significant impact on patient health and the use of health resources.

We have trained a Bayesian Network (BN) for this purpose. A BN is a probabilistic graphical model that allows the modeling of the conditional dependencies of a set of variables. It is represented as an acyclic graph where each node represents a data variable, and the edges represent the probability dependencies between nodes. By representing the relationships between variables in a graphical model, BN provides a formal communication language that is easy for humans to read, making it inherently “explainable-by-design” [25]. Systems based on BN can model complex relationships between variables when conditions of causality and conditional independence are involved. This is of great importance in the clinical decision-making process, where understanding the relationships between variables can guide more informed and accurate decisions. Additionally, the joint probability distributions can be updated if new evidence is available using Bayes’ theorem. This means that the model can adapt and improve over time as new data are collected. 

We were therefore able to model existing medical evidence and suggest potential cause/effect relationships between clinical variables and hospital admission. This can help doctors better understand the factors that influence the need for hospital admission for patients with COVID-19 and therefore make more informed and effective decisions.

We then evaluated whether the predictive performance of the BN was comparable to that of a widely used but less explainable ML model, e.g., Random Forest. Random Forest (RF) is a widely utilized machine learning technique that constructs multiple decision trees and combines their predictions to make more accurate and robust classifications. Despite being composed of explainable-by-design decision trees, elucidating the predictions of RF can be challenging due to its inherent complexity. In fact, to achieve good performance, RF usually combines at least 100 trees [26]. Therefore, RFs are commonly perceived as black box models [14,27,28], and various methods have been developed to interpret them [29,30]. However, these different approaches are not inherently incorporated into the RF’s structure itself by design and need to be implemented as an additional layer applied after predictions.

We also tested the generalization ability of the ML models across different waves of the pandemic to gauge any potential decline in ML performance over time.

## 2. Materials and Methods

During the “first wave” of the COVID-19 pandemic in Italy (from March 2020 to May 2020), we gathered data from 660 COVID-19 patients treated at the Fondazione IRCCS Policlinico San Matteo hospital in Pavia, an excellent center that is known to have successfully treated the first diagnosed COVID-19 patient in western countries. Half of these patients were hospitalized, while the remaining showed a better prognosis and were treated at home. For each patient, we collected information about age, gender, clinical features, such as C-reactive protein (CRP), symptoms, such as cough and breathing difficulties, and the presence of comorbidities, such as hypertension, cardiovascular diseases, and cancer (Table 1). 

**Figure 1:**
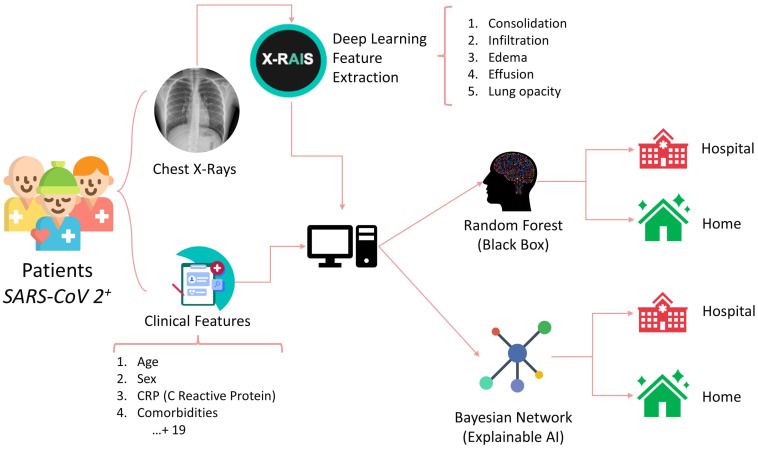
Graphical representation of patient’s medical data gathered and deep learning information used to estimate hospitalization or at-home management. Data are processed by Random Forest and Bayesian Network algorithms, determining the appropriate treatment option.

A Deep Learning algorithm was used to extract features from chest radiographs (CXR) images through the X-RAIS platform, developed by Reply™. X-RAIS is a Deep Network able to analyze different types of medical images and to extract relevant information for diagnosis. In our context, X-RAIS transformed the CXR image of each patient into 5 numerical clinically relevant features: Consolidation, Infiltration, Edema, Effusion, and Lung Opacity. These 5 features, together with 19 other clinical features, represented the input of an ML model that would predict whether a patient should be hospitalized (class 1) or not (class 0) [Figure 1]. We randomly selected 90% of the patients as a training set. The remaining 10% of patients were kept for testing and selecting the best-performing model. During the “third wave” (from March to May 2021), 462 additional patients were triaged in our Emergency Department, and 68% of them were hospitalized. This dataset is based on the ALFABETO (ALl FAster BEtter TOgether) project, whose aim is to develop an AI-based pipeline integrating data from diagnostic tools and clinical features to support clinicians during the triage of COVID-19 patients [31]. The third wave set was exploited as an additional, independent test validation set. 

The technical aspects of the current work are detailed in a previous project by our team, upon which this work is based [32].

**Figure 2:**
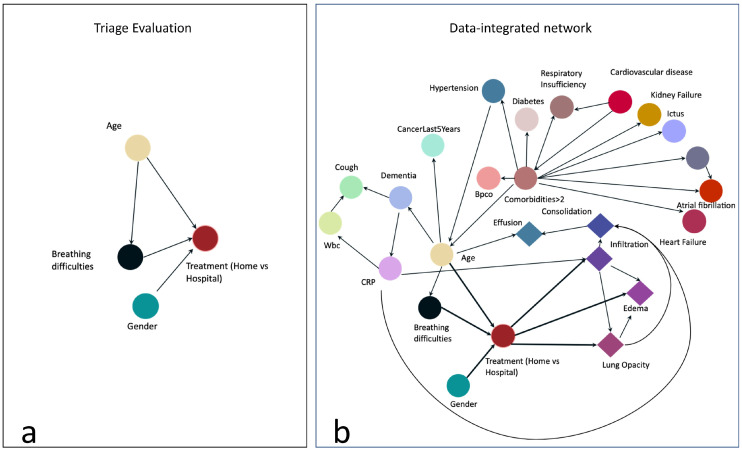
The left graph (**a**) includes relationships between some variables that represent the reasoning process of clinicians during emergency triage. The right graph (**b**) is the result of enriching it through the hill climbing search algorithm with the remaining collected variables.

To implement the BN, we first designed a graph based on our pre-existing knowledge and experts’ advice. This graph contains relationships between a few variables that may represent the clinician’s reasoning process during triage. The graph is represented in Figure 2a: the node labeled “Treatment (Home vs. Hospital)” is the target node representing our outcome of interest, i.e., whether the patient should be hospitalized or not. To make this decision, we assumed that the clinician would evaluate at least the age, the gender (male patients are more likely to incur more severe consequences from the infection), and whether the patient had breathing difficulties. The target node depends on these 3 variables, and we also assumed a direct dependency between age and breathing difficulties. We then automatically enriched the structure of this graph with the remaining collected variables by using the hill climbing search algorithm applied to the training data: starting from the constraints represented in Figure 2a, this method implements a greedy local search and performs single-edge manipulations that maximally increase a score of fitness. The search terminates once a local maximum is found [33]. 

The resulting graph is shown in Figure 2b: notably, the Boolean feature indicating whether the patient has more than 2 comorbidities (“ComorbiditiesGreaterThan2”) is explicitly linked to comorbidity nodes, such as the presence of cancer or cardiovascular diseases. Interestingly, the outcome is not directly linked to the node “ComorbiditiesGreaterThan2”, but it can be linked to the presence of comorbidities through the patient’s age. Some DL features directly depend on the target node. BN is implemented in Python 3.7, using the bnlearn package. As a preprocessing step for the BN, we transform all continuous features (such as CRP or age) into categorical attributes, based on statistics, such as median and quartile, computed on the training set. Along with the BN, we trained a Random Forest model. Random Forest is a widely applied supervised model, showing high accuracy in a variety of different tasks applied to tabular data [34,35,36]. For RF training and prediction, preprocessing of the data, such as prior categorization or normalization, was not required and therefore not implemented.

On the first test set (collected during the first wave, therefore coming from the same population of the training set) and on the third wave test set (when new variants, treatment protocols, and hospital facilities appeared), we evaluated several performance metrics. As True Positive (TP), we defined the number of hospitalized patients correctly classified by the model, while as True Negative (TN), we considered the number of patients treated at home correctly classified. The False Negative (FN) represented the number of hospitalized patients incorrectly classified as “treat at home”, while as False Positive, we indicated the number of patients treated at home that were incorrectly classified as “hospital”. In particular, we computed the Area Under the Curve (AUC) and Precision-Recall Curve (PRC), the Accuracy (as the proportion of correctly classified patients over the total number of patients), the Precision (as the ratio between the TP and the sum of TP and FO), the Recall (as the ratio between the TP and the total number of “hospital” patients), the sensitivity (as the ratio between the TN and the total number of “home” patients), and the F1 score (harmonic mean between specificity and recall). These metrics allowed us to evaluate the performance of the models under various aspects. In particular, the F1 score was selected to detect potential misclassification biases towards the “Hospital class”, as it can be more informative under imbalance data problems in comparison with Accuracy in binary classification [37].

## 3. Results

Herein we report the predicted performance of the BN in Figure 2b, whose structure is based both on expert evidence and data. The simplest network, only based on evidence (Figure 2a), shows good recall (i.e., the ability to correctly classify hospitalization) on the first test set (85%) but low specificity (around 20%), and for this reason, it was excluded from the analysis. We trained and tested three additional models: a regularized Logistic Regression, Gradient Boosting, and Random Forest (RF). We show the performance of the RF only, since it outperforms the other two models on test data. Table 2 reports BN and RF classification performance on 66 patients of the Test set, in terms of various metrics, such as Area Under the ROC Curve (AUC) and Area Under the Precision-Recall Curve (PRC), described above. On the relatively small test set, the BN showed higher performance in comparison with the RF for all the metrics of around 5%.

To test whether the error rates of the two approaches are significantly different, we apply McNemar’s Test [38], a statistical test for paired nominal data. In the context of AI/ML, McNemar’s Test can be used to compare the predictive performance of two models on the same test set. The *p*-value is 0.6, and we cannot reject the null hypothesis, i.e., the two classifiers have the same error rates. In Table 3 we can observe the prediction ability on the second test set, composed of third wave patients, where RF has slightly higher performance. Also, in this case, the *p*-value of McNemar’s test is high (0.7). In comparison with results on the first test set, both BN and RF show lower Recall, but higher Specificity, meaning that they are more accurate in predicting the “home” class. In this case, the RF performance is slightly higher in terms of F1 score, Accuracy, and Recall. To compare the AUC, we used the De Long test [39]. The resulting *p*-value is > 0.05, indicating that we cannot reject the null hypothesis, which posits that the difference in AUC is equal to zero. 

We examined the RF feature’s importance by computing the mean decrease impurity [40]. The most important feature for classification resulted in the C reactive protein (CRP), which was also directly linked to the outcome by the BN structure learning algorithm. In RF, CRP is followed by four DL-extracted features (LungOpacity, Edema, Consolidation, and Infiltration). Interestingly, the CRP has been identified as a relevant characteristic, significantly higher in severe COVID-19 patients, from data from the 4CE consortium [41]. All the DL-extracted features, except for Consolidation, have a direct link to the outcome (1b). Age, gender, and breathing difficulties are placed in the 8th, 9th, and 10th positions. The BN structure, learned from the knowledge domain and training data, was able to convey relevant information regarding the decision process. Clinicians were therefore able to understand the BN inference process about each single specific patient, as they were able to visualize the connection between features and the contingency tables. On the contrary, the feature importance as an approach to globally explain the RF does not facilitate a thorough analysis that takes feature correlation into account.

**Figure 3:**
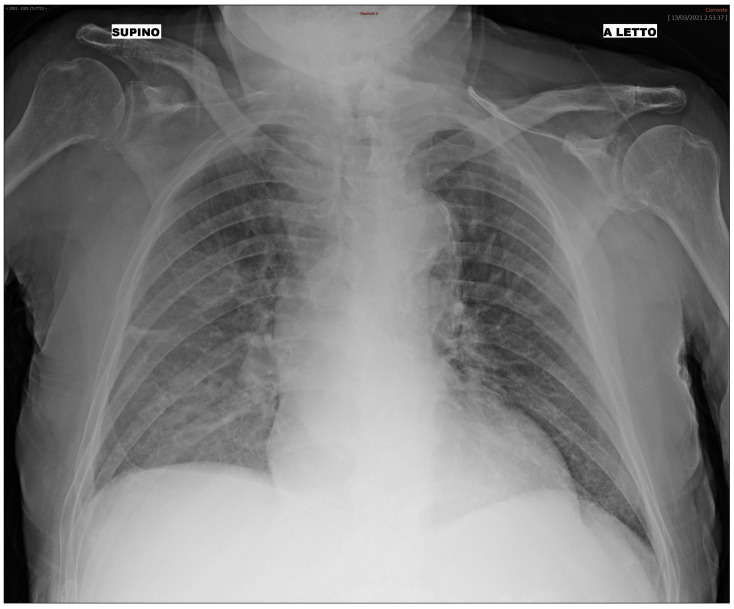
Bedside CXR image of a 92-year-old patient classified by the ML as “Hospital”.

We additionally analyzed the BN and RF performance and their misclassification errors in light of prognostic outcomes that were collected after the initial diagnosis for each patient of the third wave set. In summary, each patient included in the study (both treated at home or in hospital), underwent follow-up, and clinicians recorded the outcomes after some time from the initial diagnosis. Patients with mild outcomes were those not hospitalized or hospitalized without the need for ventilation support. Patients with moderate outcomes were hospitalized with airway pressure device support, while severe patients were those hospitalized with invasive ventilatory support or who were deceased. We analyzed misclassification errors by the BN, and we correlated it with the follow-up outcome. We found that, for False Positive patients (i.e., patients predicted “hospital” where in fact they were treated at home at the beginning), a higher percentage of severe and moderate patients were detected. In False Negative patients, most of them had a mild outcome. For instance, we present the case of a 92-year-old male patient who underwent triage during the third wave. The patient did not present with cough, dyspnea, or any significant functional impairment. The CXR image of the patient is depicted in Figure 3 and shows alteration mainly in the right lung. Both the BN and the RF incorrectly predicted the “Hospital” class, while the patient was not initially hospitalized. However, 7 days after the first triage, the patient returned to the ER with worsened symptoms and was subsequently hospitalized. This serves as an example of where the ML prediction contradicts the ground truth (i.e., the medical decision during the first triage). Nonetheless, the progression of the disease, leading to a delayed hospitalization, implies that the ML prediction might have been correct, suggesting an alternative scenario to clinicians at the time of the initial triage of the patient. The availability of the prediction from the algorithm during the first triage might suggest more caution to the clinician in weighing the chest X-ray with other factors (such as the age of the patient) and leading to more safe management even when a functional impairment is not yet present.

## 4. Discussion

In this work, we present the development of a Bayesian Network (BN) aimed at predicting whether a COVID-19 patient needs hospitalization or can be managed at home based on his clinical characteristics. In a recently published paper, Shen et al. proposed a BN-based model as a support tool for assessing the severity of COVID-19 symptoms in patients, considering epidemiology, clinical symptoms, imaging findings, and laboratory tests [42]. The performance of BN is quite similar to RF, but BN exhibits slightly higher values for all metrics. However, this study tested RF and BN on a significantly smaller number of samples.

The majority of our results have been verified by many studies that analyzed the risk factors for COVID-19-associated hospitalization [25,26,27]; specifically, the presence of at least two comorbidities was indirectly associated with hospitalization, but closely linked to age, while age > 70, male gender, high CRP blood levels, and breathing difficulties were directly correlated with an increased risk for hospitalization, as well as three distinct radiological features: Lung Opacity, Edema, and Infiltration.

In our research, both BN and RF were very effective in generalizing during the third wave of the COVID-19 pandemic, despite some changes in population variables (such as age) compared to the patients from the first wave used for training. Additionally, the predictive capacity of the BN was substantially comparable to a Random Forest approach, particularly in the third wave. The values of Precision and Specificity of the BN were even higher with respect to those of the first wave, indicating that the learned structure may not suffer from dataset shift.

While the demand for clinical decision support systems implementing ML is growing, understanding how AI algorithms correlate and classify input data is becoming increasingly important. These approaches have the ability to detect useful and hidden patterns in collected data, which can then be leveraged to support knowledge discovery and/or subsequently implement highly accurate automatic classifiers. For machine learning to be safely integrated into daily clinical practice, it is necessary to fully comprehend the classification process implemented by the algorithm. However, achieving this understanding is currently not fully feasible, as many high-performance classifiers are perceived as “black boxes”, making it difficult to grasp how the data are processed and how the output is generated.

The ability to identify subtle connections and predictions based on complex data is crucial in the context of medicine, where a swift and precise diagnosis can make a difference in a patient’s life.

To securely integrate machine learning into everyday clinical practice, a complete comprehension of the classification process of the algorithm is necessary. However, this task currently presents significant challenges. Indeed, many of the high-performance classifiers are perceived as “black boxes,” as it is difficult for physicians and healthcare providers not only to understand exactly how data are processed but also how the output is computed.

This aspect is particularly critical when AI-supported decisions directly impact patient health and well-being. The lack of transparency in AI classification processes can lead to a lack of trust in the use of such systems. Therefore, current research is increasingly focusing on interpreting and explaining AI models, seeking to make these algorithms more transparent and understandable for those using them in clinical practice. Only through a deeper understanding of how AI analyzes and interprets data will it be possible to maximize its potential in the field of medicine, ensuring both the safety and effectiveness of clinical decisions. In a clinical setting, a recent study showed that providing explanations for AI predictions can reduce the over-reliance on AI misclassification, thus allowing clinicians to spot AI errors rather than merely trust AI’s incorrect predictions [42]. Providing transparent predictions may support AI-based clinical decisions by facilitating conditions, i.e., by increasing the individual trust that technical infrastructure is in fact supporting the use of the AI-based system, which has been highlighted as an important factor in predicting the intention to use AI [43,44].

As the potential of AI is increasingly witnessed in various applications, regulatory authorities are initiating discussions on the criteria that AI-based software must meet, particularly when deployed in high-stakes applications. In 2021, the FDA introduced a draft of the Total Product Lifecycle Regulatory (TPLR) approach for AI/ML-based software serving as a medical device [45]. This approach relies on overarching principles that weigh the advantages and risks, incorporating ongoing performance monitoring and the possibility of retraining. Additionally, the European Commission is actively developing AI regulations, placing a key emphasis on trust. In 2020, they unveiled a self-assessment list aimed at ensuring the trustworthiness of AI, named ALTAI [7], which has been tailored to the medical domain (https://future-ai.eu). More recently, the transparency and explainability requirement is also reported as mandatory in the AI act, the first regulation on AI applications promoted within the European Union. Also, the World Health Organization (WHO) has released considerations and regulation of AI for health [46].

The BN allows not only the development of an explainable model by design but also the combination of known evidence of dependence on variables with information encoded in the anamnestic and clinical radiological data of patients, with the ultimate goal of assisting physicians in the decision-making process. The resulting network structure can be inspected by doctors to understand the algorithm classification process and to improve or correct correlations between data. Furthermore, we demonstrate that, in our specific problem, the BNB did not exhibit a significant decrease in performance when compared to a black box model. This finding aligns with prior research suggesting that the alleged trade-off between interpretability and performance may not hold true [24].

Considering the medical and legal aspects related to the use of artificial intelligence, as supported by Gleeson et al., “for medico-legal reasons, all contributions from non-human entities must be identifiable. In decision support systems that integrate information from imaging, medical reports, lab results, etc. for the probability of diagnoses, the recommendations change dynamically, as new information is added” [28].

Addressing the challenges of defining explainability also requires consideration of the legal context of healthcare. The European Union’s General Data Protection Regulation (GDPR) has mandated the provision of meaningful information to data subjects regarding the logic of AI algorithms, their significance, and their consequences. Although the interpretation of the GDPR is still widely debated by legal experts, the regulation primarily aims to safeguard an individual’s right to understand the decision-making process and assess the reasonableness of AI decisions. Thus, the explainability requirement does not equate to providing a causal account but rather involves elucidating the choices about the decision-making model, how data were collected, the features that were and were not considered, and the anticipated effects of the automated decision-making process. These statements suggest that explainability should consider the implications of using AI in specific clinical contexts [29].

## 5. Limitations

The main limitation of our project was the relatively small sample size, which could be increased through further retrospective studies.

The study could also be hindered by the fact that only some clinical features were included in the BN; we sought to analyze mostly variables that had already been validated by previous studies as important in the clinical evolution of the disease, but the usage of additional clinical criteria could potentially improve the width of the network or increase its correlation strength, thus giving more information to the clinician. 

Data from the second wave of the pandemic were not collected, and therefore we were not able to perform our analysis for that period. Another aspect that should be investigated is the possibility of using the model on patients from other hospitals, thus investigating the generalizability not only during time but also in space, across different hospitals, where different clinical decision protocols and patient populations may occur. As the training and test data are collected within the Italian population, our model may not generalize well to other populations.

Furthermore, an extensive comparison with other explainable models should be performed. Most importantly, we compared the explainable and black-box models in terms of performance. Future research works will involve comprehensive surveys aimed at comparing various explainable-by-design and XAI approaches in terms of usability and effectiveness in providing explanations within clinical practice. 

## 6. Conclusions

The BN enables the development of a graphically representable model, which integrates known information on the dependence of clinical variables, with information encoded in each patient’s data identified by the ML model. It also possesses predictive capabilities similar to a data-driven approach like RF, but offers greater interpretability, as it allows inspection of the BN’s structure to understand the classification process and relationships between variables.

Future works will delve into several avenues of research. This includes the exploration of new network configurations, incorporating additional layers of medical knowledge to enhance the system’s understanding and predictive capabilities. Additionally, there will be a focused effort on investigating potential causal relationships between variables, aiming to uncover deeper insights into the complex interactions within medical data.

## Figures and Tables

**Table 1:** The table provides a schematic overview of the first and third waves of COVID-19 cases, detailing demographic data, comorbidities, and symptoms presented by patients. For continuous variables (age, number of comorbidities, SatO_2_) we report mean and standard deviation (in brackets). For categorical variables, we reported the absolute frequency and the percentage.

		I Wave	III Wave
Demographic data	Men Women Age	441 (67%) 219 (33%) 66.2 (15)	266 (58%) 196 (42%) 70 (15)
Comorbidities	Number of comorbidities (average) More than two comorbidities	1.5 (1.45) 148 (22%)	0.9 (0.7) 90 (19%)
Signs and symptoms	Fever (T > 37.8 °C) Cough Dyspnea SatO_2_	153 (23%) 291 (44%) 349 (52%) 91 (7.8)	447 (96%) 129 (28%) 248 (53%) 94 (5)
Hospitalization	Yes	333 (55%)	315 (68%)

**Table 2:** Predictive performance of the Bayesian Network (BN) and the Random Forest (RF) on the Test set (66 Patients).

	AUC	PRC	Accuracy	Precision	Recall	Specificity	F1 Score
BN	0.80	0.85	0.76	0.82	0.78	0.73	0.79
RF	0.76	0.84	0.71	0.78	0.72	0.69	0.75

**Table 3:** Predictive performance of the Bayesian Network (BN) and the Random Forest (RF) during the third wave (462 Patients).

	AUC	PRC	Accuracy	Precision	Recall	Specificity	F1 Score
BN	0.80	0.89	0.71	0.89	0.65	0.82	0.75
RF	0.83	0.91	0.75	0.89	0.71	0.82	0.79

## Data Availability

The data presented in this study are available upon request from the corresponding author due to restrictions (e.g., privacy, legal, or ethical reasons).
